# Risk of Contamination of Gametes and Embryos during Cryopreservation and Measures to Prevent Cross-Contamination

**DOI:** 10.1155/2017/1840417

**Published:** 2017-08-14

**Authors:** Daniel C. Joaquim, Eduardo D. Borges, Iara G. R. Viana, Paula A. Navarro, Alessandra A. Vireque

**Affiliations:** ^1^Invitra, Assisted Reproductive Technologies Ltd., Supera Innovation and Technology Park, 14056-680 Ribeirão Preto, SP, Brazil; ^2^Department of Obstetrics and Gynecology, Faculty of Medicine of Ribeirão Preto, University of São Paulo, 14049-900 Ribeirão Preto, SP, Brazil; ^3^National Institutes of Hormones and Woman's Health, CNPq, Porto Alegre, RS, Brazil

## Abstract

The introduction and widespread application of vitrification are one of the most important achievements in human assisted reproduction techniques (ART) of the past decade despite controversy and unclarified issues, mostly related to concerns about disease transmission. Guidance documents published by US Food and Drug Administration, which focused on the safety of tissue/organ donations during Zika virus spread in 2016, as well as some reports of virus, bacteria, and fungi survival to cryogenic temperatures, highlighted the need for a review of the way how potentially infectious material is handled and stored in ART-related procedures. It was experimentally demonstrated that cross-contamination between liquid nitrogen (LN_2_) and embryos may occur when infectious agents are present in LN_2_ and oocytes/embryos are not protected by a hermetically sealed device. Thus, this review summarizes pertinent data and opinions regarding the potential hazard of infectious transmission through cryopreserved and banked reproductive cells and tissues in LN_2_. Special attention is given to the survival of pathogens in LN_2_, the risk of cross-contamination, vitrification methods, sterility of LN_2_, and the risks associated with the use of straws, cryovials, and storage dewars.

## 1. Introduction

Liquid nitrogen is a cryogenic fluid essential for in vitro reproductive technologies widely used in human in vitro fertilization (IVF), in the cattle industry (in vitro embryo production), and for livestock breeding research purposes. LN_2_ is a liquid substance produced through an industrial process by means of a fractional distillation method. Air is liquefied and then distilled in order to separate the nitrogen gas. Subsequently, nitrogen is compressed and liquefied once again, becoming ready to use [1]. Its main characteristic is the ability to maintain the ultralow temperature of −196°C, well below the freezing point of the water (0°C), making it useful for several applications. One of them is the cell cryopreservation process, used on assisted reproduction techniques to preserve gametes and embryos for the treatment of human infertility and fertility preservation issues, as well as in cryobanking of animal gametes and embryos in the cattle industry. The cryogenic temperature slows chemical and physical reactions of the biomolecules and stops the samples from degrading for future use.

Sexually transmitted infections (STIs) are of major concern to reproductive specialists. Whether in human or animal reproduction, LN_2_ is constantly being used and is a source of great concern in both medical and veterinary field, since the vast majority of commercial LN_2_ is not sterilized and thus carries great risks in the transmission and propagation of diseases. This is an issue because several microorganisms are able to survive in cryogenic temperatures found in LN_2_ and leading to its contamination and possible cross-contamination [1]. Some factors have direct impact on the risks of microbial pathogen contamination in gametes and embryos during cryopreservation, such as the integrity of the embryonic zona pellucida (ZP), the freezing method, loading and sealing of the freezing container, and the sterility of both LN_2_ and the storage container [1]. There is a potential hazard of disease transmission through cryopreserved and banked gametes and embryos in LN_2_ [1–7] and the knowledge of how to minimize or prevent contamination is necessary. Thus, this review summarizes pertinent data regarding the survival of pathogens in LN_2_ and cross-contamination, the impact of the new vitrification systems and commonly used devices, and limitations of current LN_2_ sterilization methods and offers suggestions on how to avoid the risk of cross-contamination of embryos stored in LN_2_.

## 2. Survival of Microorganisms in LN_2_ and Risk of Pathogens Transmission

Most microorganisms are able to survive in cryogenic temperatures found in LN_2_ and many are the factors that contribute to increase their resistance to low temperatures. Components in culture, freezing, and vitrification media, and semen diluents may act as stabilizers for microorganisms at low temperatures such as the used for cryopreservation [1, 8, 9]. It has been reported that a low concentration of dimethyl sulfoxide (5%) may already be effective in protecting viruses against freezing injuries [8, 9]. On the other hand, cryopreservation may reduce the bacterial population in semen. A study showed that in a concentrated suspension of* Brucella bovis* 64% did not survive to freezing and thawing procedures in culture medium without antibiotics [1, 2]. In general, bacteria have a higher tolerance to freezing and toxicity of cryoprotectants in high concentrations, while fungi are the most sensitive to these conditions. Remarkably, one study showed a 90% reduction in fungus concentration in human semen after freezing [3]. Still, there are reports of fungi and bacteria found in the debris of LN_2_ storage containers [5].

The presence of some of these microorganisms can be the result of room contamination during cryopreservation of semen and embryos. A study by Piasecka-Serafin [6] was the first to report the possibility of translocation of bacteria from contaminated semen inside sterile LN_2_. It was observed that 94% of the sterile samples were infected with* E. coli* and* S. aureus* after a period as short as two hours of exposure. There are also reports of* Pseudomonas *spp.*, Enterobacter cloacae, Staphylococcus sciuri, Acinetobacter calcoaceticus, *and* Flavobacterium* spp. introduction in LN_2_ through contaminated semen that was cryopreserved and used in in vitro fertilization [1].

**Figure 1:**
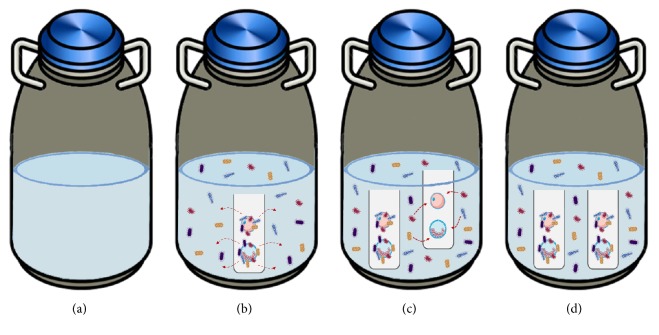
Illustration showing the process of cross-contamination in germinal tissue storage. (a) Container with “pure” LN_2_, without microorganisms. (b) Contaminated samples inserted into the container resulting in LN_2_ contamination. (c) Samples without microorganisms inserted in the contaminated container. (d) Contamination of samples that were not contaminated.

The transmission of pathogens between cryopreserved cell samples during storage in LN_2_, also called cross-contamination, poses a potential risk in assisted reproduction techniques. Basically, when a contaminated sample is placed in clean LN_2_ (free of contaminants) it will contaminate the LN_2_ and then the LN_2_ will spread the contamination to other samples stored inside the container (Figure 1). The risk of transmission of viral pathogens with significant clinical impact such as hepatitis B virus (HBV), herpes simplex, adenovirus, and papillomavirus to patients throughout the dermatologic practices of direct exposure to LN_2_ has been previously addressed [10, 11]. However, safety of cryopreserved tissue/germ cells has emerged as an important topic for ART after the discovery of a case of human hepatitis B transmission via bone marrow transplants cryopreserved in LN_2_ [12]. Noteworthy, no study to date has investigated the risk of transmission of Zika virus (ZIKV), which may be present in semen for a long period of time and cause teratogenicity [13, 14].

In a study performed in an assisted reproduction clinic, researchers evaluated semen and embryo samples stored in LN_2_ for 6 to 35 years and showed a potential increase in the risk of sample contamination through contaminated LN_2_ [5]. Forty samples were analyzed and 32 different types of bacteria were found [5]. Of these 40 samples, 14 had more than one type of microorganism (35%), the most common being* S. maltophilia* found in 10 samples. This study assessed the effects of the bacterium* S. maltophilia* on sperm and embryo and the results were alarming since it was verified that this pathogen causes a detrimental effect on sperm motility and embryonic development [5]. Of all LN_2_ samples, 69% had bacterial contamination (62% of semen and 35% of embryo samples). This study also used semen samples infected with BVDV and BHV-1 viruses and placed them in three containers with control uninfected semen and embryo samples. Control samples were not contaminated and no viruses were found in LN_2_. Inversely, another study has shown that viral contamination through LN_2_ is possible [4]. Three viral agents of animal origin (bovine viral diarrhea (BVDV), bovine herpesvirus (BHV), and bovine immunodeficiency virus (BIV)) were used to observe the potential transmission of pathogens to vitrified embryos in experimentally contaminated LN_2_. Out of a total of 83 sets of bovine embryos (3 embryos/set), 61 were exposed to both BVDV and BHV-1, and 22 were exposed to BIV, after being vitrified. The results after 3–5 weeks of storage confirmed the possibility of cross-contamination, mainly for the open system of vitrification [4]. Accordingly, 13 out of 61 sets had a positive result for BVDV or BHV-1 infection (21.3%), while none of the 22 was infected by BIV. PCR was used to determine the presence of infection in embryos [1, 4].

Due to the nature of commercial LN_2_ production system, only airborne contaminants can infect the liquid during its production; thus, it is unlikely that pathogens of major human concern, such as HIV, hepatitis B, hepatitis C, and herpes, appear in a newly produced LN_2_ [1]. In a recent study, viral sequences (HIV, HBV, and HCV) were not detected in samples of LN_2_ from containers containing oocytes and embryos from chronically infected patients; however, the same study did not rule out the risk of cross-contamination related to these viruses [15]. As a matter of interest, the risk of ZIKV transmission from gametes and embryos during ART-related procedures should be considered [16]. Noticeably, ZIKV has emerged as major health concern and fertility preservation programs were recently impacted by ZIKV spread since banned organ/tissue donations included reproductive tissues/cells such as semen and oocytes [17]. According to the latest data compiled by the European Center for Disease and Control (ECDC), a proportion of men infected with ZIKV become a source of permanent and continuing infection, posing a risk to public health [13, 18–20]. Another important point is that the maximum detection time of ZIKV in semen is 188 days [14], a period longer than that recommended for abstention of pregnancy or donation of semen or eggs after infection by ZIKV, according to the Food and Drug Administration [17]. As previously suggested the presence of ZIKV in semen and potentially in a woman's follicular fluid must be accounted for by reviewing all protocols used for gamete cryopreservation [13]. Moreover, the possibility of ZIKV survival in LN_2_ is currently not known. Zika virus contamination has never been reported in LN_2_ of human cell/tissue cryobanks but, as suggested by the British Fertility Society, the virus is likely to survive the freeze/thaw process [21]. Despite the lack of conclusive documented cases, there is a real risk of cross-contamination and, consequently, a greater spread of HIV, HBV, HVC, and ZIKV, even though the latter has not had its survival in LN_2_ proven yet. Interestingly, mycoplasma is another pathogen that, even though it cannot proliferate in LN_2_, it is able to survive in cryogenic temperatures and contaminate other samples [22]. Therefore, further studies are necessary to find more effective measures in order to prevent this kind of contamination.

## 3. Open and Closed Cryopreservation Systems

**Figure 2:**
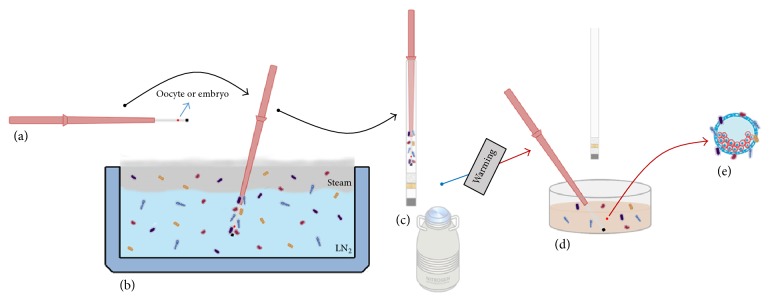
Illustration showing the open vitrification and warming system and the risk it offers for the germplasm samples. (a) Cryopreservation straw with the vitrified embryo. (b) Immersion of straw in contaminated LN_2_. (c) Straw stored in LN_2_ container. (d) Warming of the straw with the contaminated sample. (e) Contaminated germplasm.

**Figure 3:**
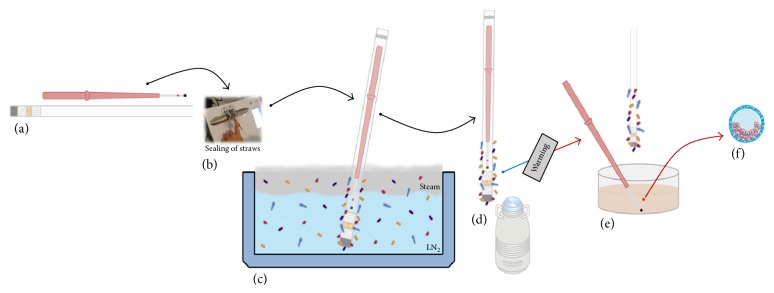
Illustration showing the closed vitrification and warming system and its low risk for germplasm samples. (a) Cryopreservation straw with the vitrified embryo. (b) The straw is covered and sealed prior to contact with LN_2_. (c) Embryo vitrification in the closed system. (d) Straw stored in LN_2_ container. (e) Straw cover is removed prior to warming, avoiding contact of the germplasm sample with the microorganisms. (f) Contaminant-free germplasm.

The recent evolution of cryopreservation techniques, cryoprotectant media, and straws/devices, allowed an expressive increase in the use of these methods. Two techniques are used in cryopreservation: slow freezing and vitrification. Pioneering studies on mammalian embryo freezing were reported in 1972 [23, 24]. Regarding the cattle industry, a successful method of freezing and thawing bovine embryos in a plastic straw followed by a one-step dilution of cryoprotectant within the straw was described in 1984 by Leibo [25]. Methods for slow, controlled-rate freezing of oocyte and early stage embryos have also greatly contributed to technological advances in cryopreservation [26–28]. Subsequently, vitrification for embryo cryopreservation was developed by Rall and Fahy in 1985 using the mouse as a model [29]. Vitrification of human embryos was introduced by Mukaida et al. in 1998 [30]. In 1999, Kuleshova et al. [31] reported the first live human birth following oocyte vitrification. Nowadays, slow freezing is more commonly used for human sperm while vitrification is applied for oocytes. Both techniques are used for human embryos with similar results [30–32]. Regarding vitrification, even though its experimental status has just recently been lifted and worldwide data is difficult to come by, it is estimated that thousands of babies have already been born as a result of this technology [33]. Today, different vitrification kits are commercially available, they differ in relation to the solutions/formulations and devices they use. There are two vitrification systems widely used for gametes and embryos: Open Pulled Straw (Cryotop®—CryoTech Lab, Cryoloop) and Closed Pulled Straw (Cryotip®—Irvine Scientific) [34, 35]. One of the first studies testing the closed “straw-in-straw” method for vitrification reported a hundred percent survival and embryo development rate, which was the same for single-straw and controls [35]. Nevertheless, vitrification presents an inherent risk to LN_2_ contamination, especially when using open systems (Figure 2), in which the straw with cryoprotectants and the genetic material is immersed directly into LN_2_. This “unprotected” method of vitrification raises the risk of contamination, but the results seem superior when compared to the closed system [36]. A survey carried in IVF centers [1, 37] where embryos and semen were placed in closed cryotubes reported cryotube explosion in 70% of samples; in 72% the presence of LN_2_ in the internal part of the tube was observed, and 80% reported problems with broken or unsealed straws. Currently, the use of a new closed system (CBS VIT HS-IMV) has been proposed for vitrification (Figure 3). Some studies showed that the use of this system had smaller rates of oocytes and embryos survival after warming than those currently obtained with the open systems [38–40]. Moreover, results obtained by De Munck et al. [41] in a current prospective controlled trial clearly failed to show the superiority of the open CryotopSC device over the closed CBSvit device. However, there is a lack of conclusive, comparative studies to demonstrate if this new system is indeed effective. We found only one article showing comparative results, where the survival rates of vitrified oocytes were similar (CBS VIT IMV = 93.7% and Cryotop SC = 89.9%) [41]. In fact, there are still two different lines of thoughts: one argues that the use of the open system has better results and an acceptable risk of contamination, especially considering that closed systems might also present contaminations risk at some level. And the other states that both systems have similar results while the closed system is significantly safer than the open system. To date, most clinics still use the open vitrification system.

## 4. Storage in LN_2_ Vapor: Could It Be a Better Alternative?

**Figure 4:**
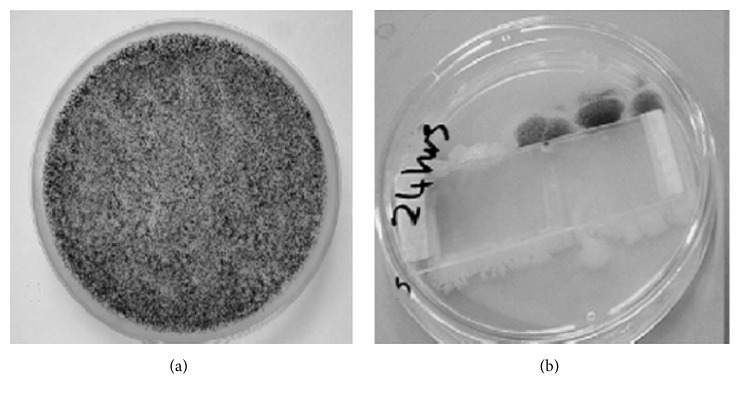
(a) Culture dish showing contamination with* S. minor* in programmable LN_2_ freezer. (b) Contamination from the vapor phase of a dry shipper, which was stocked with LN_2_ contaminated with* S. minor*.

The use of LN_2_ vapor has been proposed as a safer alternative for storing cryopreserved samples. Programmable freezers that work with vapor and dry shippers (special cryogenic storage dewar designed for transportation) were recommended for assisted reproduction clinics in order to reduce the risks of contamination. However, some studies reported that this system not only is not able to maintain the expected standard temperatures but also failed to prevent microbiological growth (Figure 4), allowing mobility of microorganisms between infected samples [2, 6, 35]. The cause for this is the formation of small ice crystals with high electrostatic charge when the water vapor in the air has contact with LN_2_. Those crystals capture microorganisms in the air and then drop into the container, collecting at the bottom of the container [2]. A study carried out with equipment working with LN_2_ vapor (Programmable Freezer and Dry Shipper) showed, through microbiological tests, that contaminant particles can be transmitted via LN_2_ vapor [2].

### 4.1. Deposit of Sediments in LN_2_ Containers and Their Potential Risk of Sample Contamination

**Figure 5:**
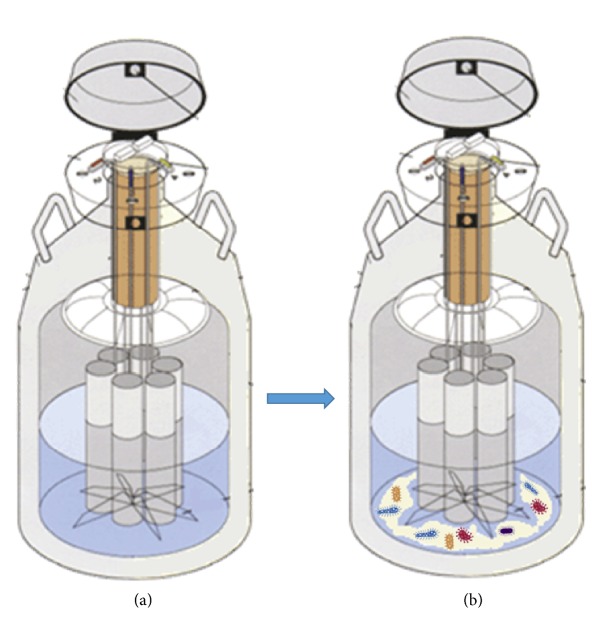
Comparison between new and used cryogenic storage dewars. (a) New dewar, without sediment. (b) After some time of usage, accumulation of sediments occurs agglomerating microorganisms in the bottom of the container.

Liquid nitrogen containers are used to store cells and tissues samples for many years impeding the possibility of proper cleaning, since exchanging samples from one container to another would offer risks like the variation in temperature, possibly impairing the viability of the samples. While this storage process is fundamental for breeding techniques, it is also a potential risk factor, since accumulated ice sediments in the container can retain bacteria, fungi, and debris in general [3]. Ice deposition occurs in two ways, either by the formation of ice in the atmosphere on the container when it is opened or by the formation of ice in the coldest parts of the container [3] (Figure 5).

A study conducted in an assisted reproduction clinic analyzed three containers that were used for 7, 12, and 15 years. The researchers let the ice pellet defrost and performed a microbiological test [3].

The results pointed out the presence of bacteria in more than 3.3% of the evaluated samples, fungal filaments were observed in 9 of 10 samples, and yeast was found in only one sample [3]. A large diversity of microorganisms was identified in the sediment from the different containers, including* A. baumannii* and* C. luteola* that may cause nosocomial infection in humans. It is important to note that the degree of contamination may not depend on the number of years the container is being used [3]. A retrospective study on a LN_2_ container that was used for 35 years revealed several contaminants (bacteria and fungi) in the LN_2_ sediment [1, 5]. These findings show the potential risk of contamination that the storage of cells and tissues brings about the assisted reproduction techniques and also the need for safer procedures for the conservation of germplasm and samples in general in LN_2_ containers.

## 5. Is It Possible to Prevent Contamination?

Unfortunately, there are few effective and practical sterilization methods available, despite the risks of microbial contamination carried through LN_2_ described in previous sessions. Even commercial distributors of LN_2_ usually do not have an effective cleaning system. On the other hand, the contact between cryopreserved samples and LN_2_ is not avoided in most devices used for vitrification. The main challenge is therefore combating the pathogens in larger volumes of LN_2_ and in the containers where it is stored as well as developing or optimizing vitrification devices to ensure hermetical cryostorage of oocytes and embryos after vitrification.

### 5.1. UV Sterilization

Ultraviolet (UV) light has been applied as a way of LN_2_ sterilization. Studies have shown that UV sterilization in small volumes of LN_2_ can be effective against bacteria, viruses, and fungi [42, 43]. A study by Parmegiani and his team [44] demonstrated that sterilization of LN_2_ with UV light has satisfactory results without causing an adverse effect on the competence and development of vitrified oocytes. In this study, MII oocytes were collected from 31 patients, totaling 168 thawed oocytes, with 151 oocytes surviving (89.9%). From those, 126 oocytes were submitted to ICSI (Intracitoplasmatic Sperm Injection), obtaining a fertilization rate of 88.3% (107/126), 71% (76/107) of cleavage, and 26.3% (20/76) of embryos (grade 1). In the oocytes from the fresh group, the results were 88.3% (106/120), 72.6% (77/106), and 33.8% (26/77), respectively. Thus, no significant differences between oocytes vitrified using UV-sterilized LN_2_ and fresh oocytes were observed [44].

In another study by the same authors [45], two sterile 500 ml LN_2_ containers were contaminated with bacteria (*P. aeruginosa*,* E. coli,* and* S. maltophilia*) and a fungus* (A. Niger)*. Then, 232 straws (Cryotop-Kitazato) with human oocytes and embryos were immersed in contaminated LN_2_, of which 182 were infected with bacteria and 50 with fungi. Subsequently, 142 samples were tested using standard bacteriological methods in order to certify that they were free of contamination. These samples were split into two groups (no wash and 3 sequential washings with UV-sterilized LN_2_). In the group that did not receive the washing procedure, 92 of the 117 (78.6%) samples exposed to bacteria and 25 of the 25 (100%) samples exposed to the fungus were contaminated. In the group submitted to of three washing procedures, no contamination was detected (bacterium: 0/65; fungus 0/25). The results were very satisfactory, demonstrating efficacy in the sterilization of LN_2_ with UV light [45].

A downside of this method is the formation of ozone caused by UV rays. The great oxidizing power of the ozone makes it a threat for oocytes and embryos. However, it has already been confirmed that the formation of ozone is insignificant due to the environment where the UV light is launched [44]. Liquid nitrogen is virtually free from O_2_ and ozone is formed only from the breakdown of oxygen molecules by the action of ultraviolet radiation when separate atoms combine with other oxygen molecules [44].

Recent studies show that the use of UV light in a suitable dose of radiation, in a small volume of LN_2_, can prevent the growth of all types of microorganisms, such as hepatitis B virus (dose of 8000 *μ*W/cm^2^) and* Aspergillus niger* (dose of 330,000 *μ*W/cm^2^). Most viruses can be inactivated at a dose of 200,000 *μ*W/cm^2^, but it is known that the ZIKV showed a somewhat increased resistance to UV when compared to dengue virus [46], for example. So the effectiveness of this approach for handling ZIKV in cryopreservation procedures must be investigated in order to find the precise UV dose for its complete inactivation [46]. To access the complete list of microorganisms susceptible to germinal ultraviolet UV see https://ultraviolet.com/microorganisms-deactivated/.

### 5.2. Sterilization of LN_2_ by Means of Filtration and New Methods

Filtration was proposed as an alternative to sterilize LN_2_ but few studies on this subject have been published. In one of those studies a 0.22 *μ*m polytetrafluoroethylene (PTFE) filter and* Brevundimonas diminuta,* as a contaminant, were used [47]. Prior to the filtration process, the PTFE filter was submitted to the autoclave sterilization process. Testing of the filter is required to ensure that the membrane remains intact and has the ability to retain bacteria. Before and after each sterilization cycle the filter was evaluated. The study used extreme temperatures, high pressures, high flow rates, and high concentration of bacteria. The results validated the efficacy of LN_2_ sterilization. The 0.22 *μ*m PTFE filter was effective for* B. diminuta* removal even after extreme conditions.

**Figure 6:**
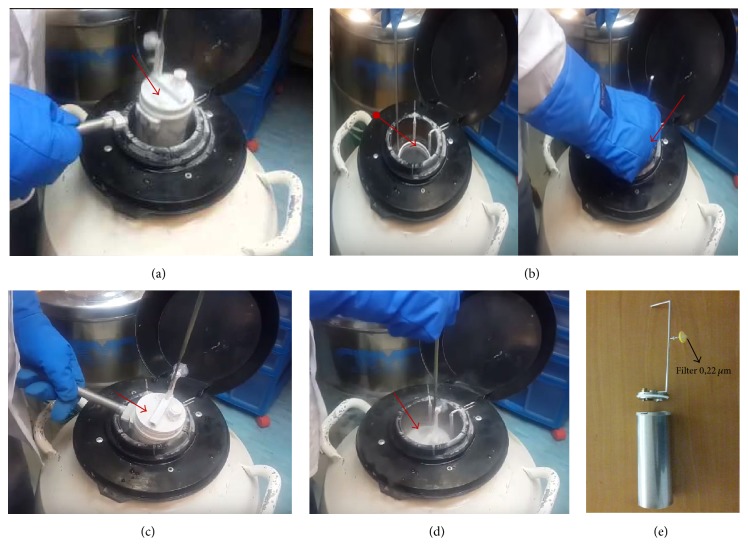
Sequence of images showing how the Esther system works. (a) Suspension of the Esther to remove its cap. (b) Insertion of non-Esther samples without it remaining in the LN_2_ container exposed to the environment. (c) Close of Esther. (d) Esther immersion (with as samples) in the LN_2_ cylinder. (e) Image showing Esther's full format.

A new method for storing cells and gonadal tissue samples at low temperatures (−196°C) in a clean environment was recently proposed [48]. The Clean Liquid Air (CLAir)® in conjunction with Esther® is a benchtop equipment for the production of sterile air using LN_2_. Esther is an insulation device adapted in the containers of the LN_2_ storage dewar (Figure 6). This device has a 0.22 *μ*m filter and the sample is exposed only to sterile LN_2_. Tests of temperature stability, freezing rates, embryo development, survival of vitrified oocytes (MII), and contamination rates showed no significant differences when compared to the commonly used method.

### 5.3. Sterilization of LN_2_ Storage Dewars and Dry Shippers

Any cleaning product can be used for the disinfection of LN_2_ storage dewars as long as it does not react with aluminum. In general, its manufacturing companies recommend use of 10% of common detergent in H_2_O. Other products can be used, such as a solution with 3% to 6% hydrogen peroxide and 37% alcohol, rinsing with water [1]. The problem is that the container must be empty and this can put the samples at risk. The International Embryo Technology Society (IETS) and the World Organization for Animal Health recommend cleaning with a frequency of six months to one year [1].

Dry Shipper is another type of container used to store germ cells. Despite the use of LN_2_ to fill it, its inside is built with spongy material, which absorbs and conserves liquid nitrogen. It is precisely the way it is built that makes its sterilization so complicated. Microbiological contamination of two containers (Dry Shipper) with different absorbent membrane types (hydrophobic and nonhydrophobic) was identified and then biocides were used for the disinfection [49]. This study showed that containers with hydrophobic absorbent membrane are more easily disinfected using liquid biocides. Some products, like ethanol, may cause irreversible damage to the absorbent membrane, while others are not effective for disinfecting. The use of ethylene oxide (EtO) was effective for both types of absorbent membrane. Besides its great antimicrobial power, the use of EtO also has the advantage of eliminating the use of liquid products, reducing the risk of damages to the absorbent membrane [49].

### 5.4. Hermetically Sealed Cryopreservation Devices and Cryostorage

**Figure 7:**
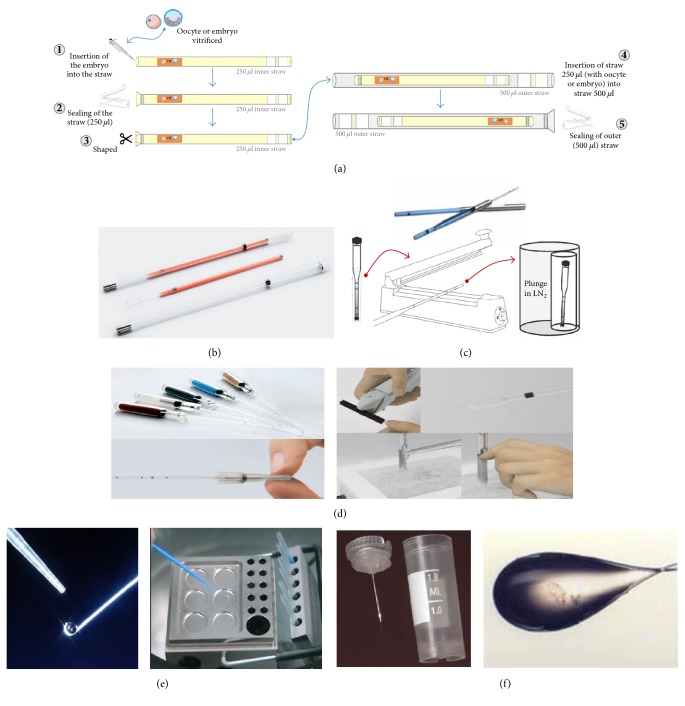
Sequence of images showing the different methods of vitrification in the “closed” system. (a) Straw-in-straw. (b) Cryotop—Kitazato. (c) Cryotip—Irvine Scientific. (d) Cryopette—ORIGIO. (e) CVM™ CryoLogic Vitrification Method. (f) Cryoloop.

The “straw-in-straw” principle or closed system has been invented for embryo cryopreservation by immersion into LN_2_ [35] and this system is still used and even expanded on safe method for cryostorage stem cells [50]. All other “closed” systems that used outer straw are modifications of the same arrangements (Figures 7(a)–7(c)). One of the current challenges on this topic is to develop cryopreservation methods and/or devices that can guarantee the lowest possible risk of contamination without interference in the success rates of the technique.

The open vitrification system is widely used in assisted reproduction, with proven success rates. However, its unprotected form allows the contact of the samples to LN_2_, exposing them to potential pathogens. This causes embryologists around the world to look for alternative methods to increase biosafety in the process, until further studies prove the effectiveness of the (fully) closed system without any contact with LN_2_. However, between these two methods, there are alternative methods that are said to be “closed” (Figure 7) but actually do not avoid direct contact with LN_2_ and/or LN_2_ vapor [51]. In many cases they are sealed after vitrification, that is, after direct contact with LN_2_ or with steam. On the other hand, they can greatly reduce the risk of cross-contamination [22, 51]. One such method is the use of a “stem” to vitrify using purified LN_2_ and the storage of this “stem” is done in a sterile container and later sealed (OPS and Cryotop) [22, 51]. Metal block vitrification (Cryologic®) was also proposed [51] as an alternative to avoid direct contact with LN_2_. In this method a metal block is submerged in LN_2_ and the vitrification occurs on the surface of the block. But, it does not prevent the contact of the sample with the LN_2_ steam and consequently the possibility of contamination. There is also another method applied in cryopreservation where a thin, narrow-walled capillary (Cryotip and Cryopette®) is used [36, 41, 45]. In this system the cell is bottled, sealed, and subsequently immersed in LN_2_. But, safety is compromised since the thawing is done in water. In addition, a decrease in the cooling and heating of the sample may occur, impairing the success rates of the technique.

The use of Cryloop [22, 51] is still reported as an alternative in which the sample is vitrified directly in LN_2_ and stored in a cryotube. Storing cryopreserved samples in this method does not prevent contact with LN_2_ and is ineffective against cross-contamination. Bielanski [22] reported that the use of cryotubes can present risks since 45% of cryotubes without O-ring and 58% with O-ring absorbed LN_2_ after 3 h of immersion in LN_2_.

Finally, another important biosafety measure is the segregation of samples [22]. Storage of contaminated or suspect samples may contaminate LN_2_ resulting in cross-contamination. It is therefore recommended that semen and/or embryo from infected or suspected donors should be quarantined, be tested, and, if contamination is found, be stored in separate containers [22]. These reports evidenced the need to improve the cryopreservation procedures, maximizing biosafety of the samples, without harming the success rates of the technique.

## 6. Final Remarks

For the safe and successful cryopreservation of gametes and embryos, the freezing method and devices must be chosen carefully to minimize the risk of disease transmission when those gametes are used for ART and fertility preservation approaches. Based on research to date there is no scientific consensus on the safety of claimed high-security closed methods in comparison with current open vitrification systems. While infectious transmission has never been observed in gametes and embryos, methods to sterilize LN_2_ were developed such as microfiltration or ultraviolet (UV) radiation. However, until more robust evidence regarding the risk of disease transmission and reviewed guidelines are available, the implementation of rational measures to minimize the theoretical danger of infectious organisms transmission seems pertinent. Despite unclarified issues, there are a set of safer practices that can be implemented in order to minimize the risk of contamination during cell cryopreservation and long-term storage. Advances in research on vitrification systems and better approaches for handling pathogens in ART/cryopreservation, including ZIKV, are needed. Specifically, cryopreservation procedures safety measures should be revised and more investments should be made in order to make closed systems more efficient. Also, new, more efficient, methods of LN_2_ and containers sterilization should be applied regularly to prevent contamination risks and damage to the stored cells. More efforts are strongly recommended in order to not only make closed systems more efficient but also review cryopreservation procedures and consider the application of the new LN_2_.
